# Morphogenesis underlying the development of the everted teleost telencephalon

**DOI:** 10.1186/1749-8104-7-32

**Published:** 2012-09-18

**Authors:** Mónica Folgueira, Philippa Bayley, Pavla Navratilova, Thomas S Becker, Stephen W Wilson, Jonathan DW Clarke

**Affiliations:** 1 Research Department of Cell and Developmental Biology, UCL, Gower Street, London, WC1E 6BT, UK; 2 Department of Cell and Molecular Biology, University of A Coruña, 15008, A Coruña, Spain; 3 Sars Centre for Marine Molecular Biology, University of Bergen, 5008, Bergen, Norway; 4 Developmental Neurobiology and Genomics, Brain and Mind Research Institute, Sydney Medical School, University of Sydney, Camperdown, NSW, 2050, Australia; 5 MRC Centre for Developmental Neurobiology, King’s College London, Guy’s Hospital Campus, London, SE1 1UL, UK

**Keywords:** eversion, olfactory bulb, ray-finned fishes, telencephalon, zebrafish

## Abstract

**Background:**

Although the mechanisms underlying brain patterning and regionalization are very much conserved, the morphology of different brain regions is extraordinarily variable across vertebrate phylogeny. This is especially manifest in the telencephalon, where the most dramatic variation is seen between ray-finned fish, which have an *everted* telencephalon, and all other vertebrates, which have an *evaginated* telencephalon. The mechanisms that generate these distinct morphologies are not well understood.

**Results:**

Here we study the morphogenesis of the zebrafish telencephalon from 12 hours post fertilization (hpf) to 5 days post fertilization (dpf) by analyzing forebrain ventricle formation, evolving patterns of gene and transgene expression, neuronal organization, and fate mapping. Our results highlight two key events in telencephalon morphogenesis. First, the formation of a deep ventricular recess between telencephalon and diencephalon, the anterior intraencephalic sulcus (AIS)*,* effectively creates a posterior ventricular wall to the telencephalic lobes. This process displaces the most posterior neuroepithelial territory of the telencephalon laterally. Second, as telencephalic growth and neurogenesis proceed between days 2 and 5 of development, the pallial region of the posterior ventricular wall of the telencephalon bulges into the dorsal aspect of the AIS. This brings the ventricular zone (VZ) into close apposition with the roof of the AIS to generate a narrow ventricular space and the thin tela choroidea (tc). As the pallial VZ expands, the tc also expands over the upper surface of the telencephalon. During this period, the major axis of growth and extension of the pallial VZ is along the anteroposterior axis. This second step effectively generates an everted telencephalon by 5 dpf.

**Conclusion:**

Our description of telencephalic morphogenesis challenges the conventional model that eversion is simply due to a laterally directed outfolding of the telencephalic neuroepithelium. This may have significant bearing on understanding the eventual organization of the adult fish telencephalon.

## Background

The mechanisms underlying CNS regionalization and specification of neuronal identity are remarkably well conserved across all vertebrates [1-3], yet there is huge diversity in the mature morphology of brains of different species [4,5]. In addition, the mechanisms underlying differential morphogenesis and growth regulation of homologous CNS domains between species are not understood; indeed this topic has barely been studied. The region of the brain that shows the greatest variation in its morphology is the telencephalon, and this region is probably least well understood in terms of homology between vertebrate classes [6-11]. This is particularly true of the brains of ray-finned fish, which are markedly different from those of other vertebrates [11-13]. Since the zebrafish brain has become an important experimental model for vertebrate brain development, a deeper understanding of its morphogenesis is required, to better define its homologies and functional organization.

**Figure 1:**
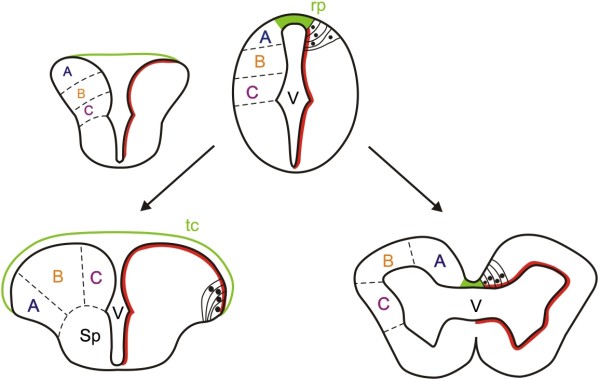
**Conventional model for eversion in ray-finned fishes.** Cartoon showing the classic view for eversion in ray-finned fishes (left) in contrast with the evagination of the telencephalic vesicles that occurs in other vertebrates (right). In ray-finned fishes, it is thought that the dorsal region of the telencephalic neural tube (pallium) folds over the ventral region (subpallium), stretching the dorsal roof-plate region of the neural tube (green) to form the tela choroidea. This would also relocate some ventricular cells to the dorsal telencephalic surface (red dotted line) and cause medial to lateral rearrangements of pallial regions (compare the location of regions A, B, and C between everted and evaginated telencephali). Modified from [16]. rp, roof plate, sp, subpallium, tc, tela choroidea, v, ventricle.

In ray-finned fishes (and partially also in the coelacanth [14]) the forebrain contains two solid telencephalic lobes, separated by a T-shaped ventricle (Figure 1). The dorsal surface of the telencephalon is thus a thin tela choroidea that covers the T-shaped ventricular space. In non-ray-finned fishes and other vertebrates, the telencephalon consists of two hollow hemispheres, each surrounding an inflated lateral ventricle (Figure 1). The telencephalon of ray-finned fishes is referred to as ‘everted’ while the more conventional vertebrate telencephalon is described as ‘evaginated’ [15].

The current widely held view of how eversion occurs is based largely on extrapolation from the morphology of the telencephalon in adult and larval fishes [13,15,17-22], but no detailed experimental analyses of telencephalic morphogenesis have been performed. According to the current view, starting from a simple hollow tube in early development, eversion is thought to consist of an outward bending of the lateral walls of the telencephalon, resulting in a lateral outfolding of the dorsal region of the neural tube, such that the dorsal telencephalon (pallium) folds laterally over the ventral telencephalon (subpallium) [5,11,23] (Figure 1). By this process, the initially narrow telencephalic ‘roof plate’ expands laterally over both left and right telencephalic lobes to become a thin tela choroidea covering the everted telencephalic surface and enclosing the ventricular space (Figure 1). However, the positions of the pallial areas in the adult telencephalon, especially at the caudal pole of the pallium, do not fit with some of the predictions based on the hypothesis of simple mediolateral outfolding [9-11,19,24]. Thus, modifications of the classical model have been proposed, to explain the location of posterior pallial areas in the adult brain [9,10,19,24]. Yamamoto and collaborators [10], for example, suggest that there must be major differences in the morphogenetic movements of eversion along the anteroposterior axis, and they imply that mediolateral folding at the posterior pole is more complex than at the anterior pole. These new hypotheses for eversion have yet to be tested in the embryo.

Understanding the development of the two fundamentally different telencephalic morphologies - everted and evaginated - will be useful not only for deciphering common and divergent mechanisms in CNS morphogenesis, but also for understanding and assigning homologies between the neuronal groups and regions in fish and other vertebrates. Therefore, we have performed a detailed analysis of early telencephalon morphogenesis in the zebrafish, a teleost with an everted telencephalon. Our data show two key steps in telencephalon morphogenesis. The first step involves the generation of a deep ventricular sulcus between telencephalon and diencephalon (the anterior intraencephalic sulcus or AIS), which creates a posterior telencephalic wall and shifts the most posterior pole of the dorsal telencephalon to a more lateral position. This is followed by a large expansion of the pallial territory, effectively elongating the dorsal telencephalon along the anteroposterior axis so that the ventricular surface of the pallium bulges into the dorsal aspect of the AIS. During this phase, the roof of the AIS expands forward over the telencephalon to form the tela choroidea. Our data do not support the widely held model that the everted phenotype results from an outward bending of the lateral walls of the telencephalon.

## Results

### Formation of the anterior intraencephalic sulcus (AIS) is the first step in telencephalic lobe morphogenesis

**Figure 2:**
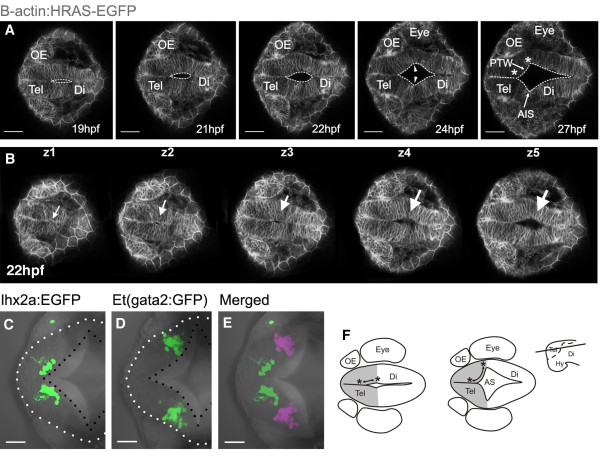
**Formation of anterior intraencephalic sulcus (AIS) and posterior wall of the telencephalon. A**. Time-lapse sequence of a single z-level of a Tg(β-actin: HRAS-EGFP) ^vu119^ embryo. The midline lumen opens out at the telencephalic-diencephalic boundary to form the AIS and the posterior wall of the telencephalon (ventricular surface between asterisks). Inflation of the ventricular space leads to a lateral protrusion of the telencephalon in its caudal region (arrowheads). **B**. Five z-levels at a single timepoint showing opening of the AIS (arrowed) is initiated deep (z5) from the dorsal surface (z1). **C**. Cells of the olfactory bulb, revealed in Tg(−10lhx2a:EGFP)^zf176^ line, lie medially and just in front of the posterior telencephalic wall at 30 hpf. **D**. Pallial cells in the Et(gata2:GFP)^bi105^ line lie laterally, just in front of the posterior telencephalic wall at 30 hpf. **E**. Olfactory bulb [Tg(−10lhx2a:EGFP)^zf176^] and pallial cells [Et(gata2:GFP)^bi105^] in C and D superimposed to show relative positions (pallial cells pseudocoloured turquoise). **F**. Diagram to illustrate how generation of AIS folds posterior telencephalon to a more lateral location. Dorsal views, anterior to the left. Scale bars: 100 μm in A and B and 50 μm in B to E. AIS: anterior intraencephalic sulcus; Di: diencephalon; Hy: hypothalamus; OE: olfactory epithelium; PTW: posterior telencephalic wall; Tel: telencephalon.

The T-shaped telencephalic ventricle of teleosts is a hallmark of the everted telencephalon, so we first sought to understand how it acquires this shape. To do this, we analyzed forebrain ventricle morphogenesis by 4D time-lapse imaging and by visualizing neuroepithelial cell outlines using the Tg(β-actin:HRAS-EGFP)^vu119^ transgene [25]. At 15–16 hpf (hours post fertilization), the forebrain primordium is a compact mass of polarized cells (neural rod) with no lumen. The ventricular system begins to appear at around 18 hpf, starting with the opening of the hindbrain ventricle and continuing rostrally to form the mesencephalic ventricle and, finally, the forebrain ventricle (Figure 2A, Additional file 1: Movie 1). At the telencephalic-diencephalic border, a shallow outfolding of the neuroepithelium appears (Figure 2, Additional file 1: Movie 1), which is followed by inflation of the ventricle in this region to form a distinct ventricular sulcus, the anterior intraencephalic sulcus (AIS, labelled AS in [26]). Analysis of time-lapse data at different z-levels within the dorsal neural primordium shows that the AIS forms in a ventral to dorsal progression (Figure 2B, and Additional file 1: Movie 1). In addition, generation of the AIS in the dorsal neural primordium lags considerably behind formation of the more ventral and rostral optic recess that accompanies out-pocketing of the eye primordia (data not shown). Thus, although the optic recess and AIS form a continuous groove, they appear to be generated by temporally separate morphogenetic steps.

Our time-lapse data show that during formation of the AIS, the most posterior neuroepithelial cells of the prospective pallial telencephalon are displaced laterally (Figure 2A,B). To test this idea further, we analyzed expression of transgenes expressed in neural cells in anterior and posterior pallial telencephalon (see below and Methods for details of the transgenes). At 30 hpf we find that pallial neurons that express Et(gata2:GFP)^bi105^ are displaced laterally compared with the more anterior olfactory bulb neurons expressing Tg(−10lhx2a:EGFP)^zf176^. Both transgenes are expressed just in front of the dorsal rim of the AIS, with the more topologically anterior olfactory bulb (OB) expression located medial to the more topologically posterior pallial expression (Figure 2C,D,E).

By the end of the first day of development, each of the two telencephalic lobes is a roughly ellipsoidal solid, with flat medial surfaces adjacent to the narrow midline ventricle, and bounded posteriorly and ventrally by the curved ventricular surface of the AIS. So at this stage, both telencephalic lobes have a medial ventricular surface sandwiched between the two lobes and a posterior ventricular surface, which we will refer to as the posterior telencephalic wall. The AIS has a thin diamond-shaped roof dorsally.

### Morphogenesis of olfactory bulb, pallial, and subpallial territories

**Figure 3:**
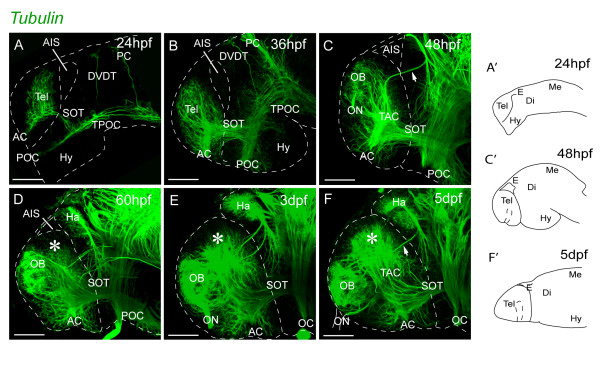
**Neuropil organization in the telencephalon between 1 dpf and 5 dpf. A**-**F**. Acetylated α-tubulin inmunostaining. The pallial telencephalic neuropil (indicated by asterisks) expands greatly posterior to the OB between days 2 and 5. The forebrain flexure places the subpallium and hypothalamus more posteriorly from 24 hpf to 36 hpf (A and B). At 2 dpf, the olfactory bulb neuropil is located close to the dorsal rim of the AIS and there is no obvious pallial neuropil. The pallial neuropil (asterisk) expands greatly posterior to the OB between days 2 and 5 (**D**-**F**). Lateral views, anterior to the left. A’, C’ and F’ illustrate the orientation of telencephalon in the related photomicrographs. Scale bars, 50 μm. AC: anterior commissure; AIS: anterior intraencephalic sulcus; Di: diencephalon; DVDT: dorsoventral diencephalic tract; E: epithalamus; Ha: habenula; Hy: hypothalamus; Me: mesencephalon; OB: olfactory bulb; OC: optic chiasm; ON: olfactory nerve; PC: posterior commissure; POC: postoptic commissure; SOT: supraoptic tract; TAC: tract of the anterior commissure; Tel: telencephalon; TPC: tract of the posterior commissure; TPOC: tract of the postoptic commissure.

To understand the morphogenesis of the dorsal telencephalon beyond 24 hpf, we analyzed the appearance and relative positions of the olfactory bulb, pallial, and subpallial territories over the first five days of development. First, we assessed the organization of axons and neuropil using the acetylated tubulin antibody (α-tubulin; [27]) from 1 dpf to 5 dpf (Figure 3). The olfactory bulb territory (OB) becomes obvious between 36 hpf and 48 hpf, owing to the characteristic organization of fibres in the olfactory glomeruli (Figure 3C). The OB initiates its differentiation in a dorsal location, close to the dorsal rim of the AIS. Until 48 hpf, the prospective pallial region caudal to the nascent OB is largely devoid of axonal labelling, suggesting relatively little differentiation in this region. However, after 60 hpf, the territory between the OB and the posterior telencephalic wall expands greatly and is occupied by neuropil (asterisks on Figure 3D-F), suggesting increased pallial growth and differentiation. In subpallial regions, the anterior commissure is located close to the rostral tip of the telencephalon at 24 hpf. From 24 hpf to 48 hpf, the anterior commissure is positioned more caudally relative to the rostral tip of the telencephalon, concomitant with the tissue movement that repositions the hypothalamus backwards beneath the midbrain (Figure 3; [1,27-29]. The origin and morphogenesis of the OB territory close to the dorsal rim of the AIS was confirmed by tracking the expression of the OB markers *lhx1a* and Et(CLG-YFP)^smb8^ (Additional file 2: Figure S1A-F) and by using Kaede photoconversion to fate map the telencephalic territory adjacent to the dorsal rim of the AIS from 24 hpf (Additional file 2: Figure S1G-J) .

**Figure 4:**
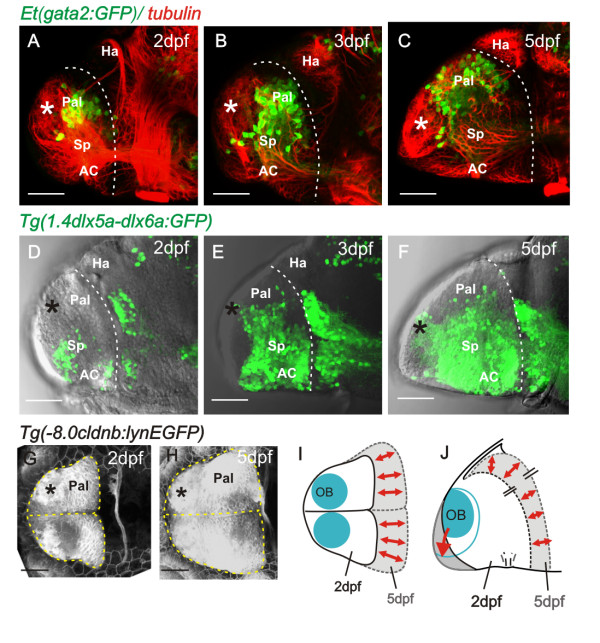
**Pallial and subpallial expansion between 2 dpf and 5 dpf. A** to **C**. Lateral views of 2, 3, and 5 day telencephali expressing [Et(gata2:GFP)^bi105^] in pallial neurons whose numbers expand into the region between the OB (asterisk) and the diencephalic habenulae. **D** to **F**. Lateral views of 2, 3, and 5 day telencephali expressing Tg(1.4dlx5a-dlx6a:GFP)^ot1^ in subpallial cells, whose numbers expand greatly between days 2 and 5. **G** and **H**. Dorsal views of telencephalon at 2 and 5 days labelled with the transgene −8.0cldnb:lynEGFP. Positions of olfactory bulbs are superimposed and the outlines of the telencephali are overlaid in I (dorsal view) and J (lateral view), to illustrate that most growth occurs along the anteroposterior axis but that the mediolateral dimensions alter relatively little. AC: anterior commissure; Hab: habenula; OB: olfactory bulb; Pal: pallium; Sp: subpallium.

Our analysis of the neuropil suggests extensive differentiation of the pallium from 2 dpf to 5 dpf. To compare pallial and subpallial differentiation over this period, we analyzed the expression of transgenes labelling pallial neuronal precursors or neurons [Et(gata2:GFP)^bi105^] and subpallial neuronal precursors or neurons [(Tg(1.4dlx5a-dlx6a:GFP)^ot1^]. At 2 dpf, the expression of the Et(gata2:GFP)^bi105^ transgene shows that only a few pallial cells have differentiated adjacent to the OB domain (Figure 4A). The number of pallial neurons intervening between the OB and the habenular region of the diencephalon then increases significantly between 2 dpf and 5 dpf (Figure 4B and C). Subpallial cells expressing the Tg(1.4dlx5a-dlx6a:GFP)^ot1^ transgene are also few in number at 2 dpf, and also expand substantially through 3 dpf to 5dpf (Figure 4D-F). These results suggest that the expansion of pallial and subpallial domains is roughly similar from 2 dpf to 5 dpf.

To assess the overall growth and form of the telencephalon between 2 dpf and 5 dpf, we used the Tg(−8.0cldnb:lynEGFP)^zf106^, which fortuitously strongly labels the membranes of all telencephalic cells (Figure 4G and H). Observations from the dorsal and lateral aspect show rather little change in mediolateral and dorso-ventral dimensions but a significant increase along the anteroposterior (AP) axis (Figure 4I and J).

Taken together, these data show that the OB initially differentiates close to the dorsal rim of the AIS and is separated by only approximately 20 μm from the dorsal diencephalon. Between 2 dpf and 5 dpf, telencephalic domains posterior to the OB grow considerably, bulging into the AIS and increasing the distance between the OB and the dorsal diencephalon.

### Expansion of the dorsal ventricular surface and morphogenesis of the tela choroidea from 2 dpf to 5 dpf

**Figure 5:**
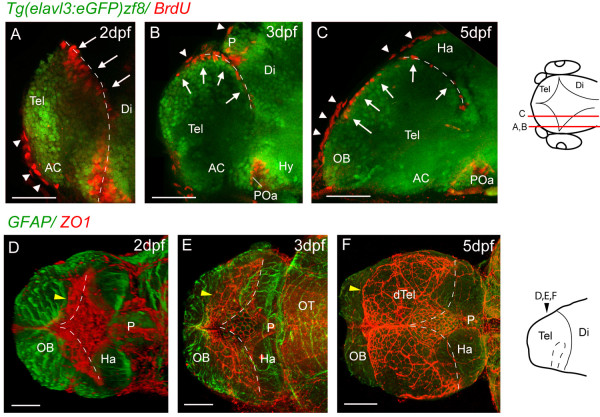
**Proliferative cells and tela choroidea expand over the upper telencephalic surface between 2 dpf and 5 dpf. A**, **B** and **C**. Parasagittal sections of BrdU-labelled (red) telencephali at 2, 3, and 5 days. At 2 days, the BrdU nuclei in the telencephalon (arrows) are seen lining the posterior telencephalic wall. By 3 days, a few nuclei are found on the upper surface of the telencephalon, and by 5 days, a more extensive area of the upper surface is lined with BrdU-positive nuclei (arrows). The more flattened BrdU-positive nuclei (arrowheads) are present in the overlying skin. **D**, **E** and **F**. ZO1 staining (red) reveals that the tela choroidea spreads over the dorsal surface of telencephalon over the same period as the proliferative cells. The diamond-shaped roof of the TDR at 2 dpf expands (yellow arrowhead) to cover the upper telencephalic surface (except for the OBs) by 5 dpf. Each image is a dorsal view created by projecting a z-stack of horizontal confocal sections. Neuroepithelium is counterstained with an anti-GFAP antibody (green)**.** Dotted lines mark the position of the posterior telencephalic wall. Schematics on the right show levels of sections and planes of view. Scale bars 50 μm. AC: anterior commissure; AIS: anterior intraencephalic sulcus; Di: diencephalon; dTel: dorsal telencephalon; Hab: habenula; OB: olfactory bulb; P: pineal; POa: preoptic area; Tel: telencephalon. Dashed lines indicate medial and posterior walls of the telencephalon.

To understand the expansion of the pallium in relation to the emergence of the everted telencephalic ventricle, we analyzed the ventricular surface from 1 dpf to 5 dpf. Between 24 hpf and 48 hpf, proliferative cells are only located in the medial and posterior ventricular walls of the telencephalic lobes (Figure 5A-D and Additional file 3: Movie 2). Between 2 dpf and 5 dpf (i.e., during the period of pallial expansion), we observe in lateral views that proliferative cells now also appear on the upper pallial surface of the telencephalic lobe in addition to the ventricular surfaces of the medial and posterior telencephalic walls (Figure 5A-C). The upper surface of the olfactory bulbs never contains proliferative cells (Figure 5C).

The expansion of proliferative cells over the upper pallial surface from 2 dpf should be accompanied by a thin epithelial tela choroidea covering these superficial ventricular cells. To assess this, we followed tela choroidea formation using the epithelial junction protein ZO1 to reveal these flattened epithelial cells. At 1 dpf and 2dpf, the prospective tela forms a roughly diamond-shaped roof over the dorsal extremity of the AIS. From 2 dpf through to 5 dpf, the area of the roof enlarges greatly along its AP dimension but relatively little along its transverse (mediolateral) dimension (Figure 5D-F), so that by 5 dpf almost all the upper surface of the telencephalon is covered by tela choroidea (Figure 5F).

The OBs do not become covered by tela; rather, the tela is attached just caudal to their posterior limit (Figure 5F). This close anatomical relation of OB to the attachment of the tela choroidea is maintained into adulthood and has been documented in other teleost brains (Additional file 4: Figure S2 and [12,30,31]).

### Fate mapping demonstrates elongation of the telencephalon along the AP axis from 2 dpf to 5 dpf

**Figure 6:**
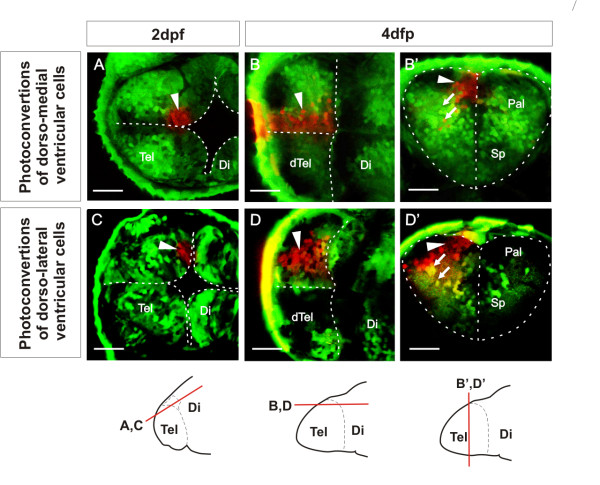
**Ventricular zone at dorsal rim of the AIS expands along the AP axis from 2 dpf to 4 dpf. A**. Single confocal z-level in the horizontal plane shows ventricular cells photoconverted (arrowhead) in a medial location of the dorsal rim of the AIS at 2 dpf. **B**. By 4 dpf, a similar horizontal plane close to the upper (now ventricular) surface of the telencephalon shows that these cells have generated an anteroposteriorly expanded column of cells. B’. A transverse plane through these cells shows that most photoconverted cells are ventricular zone cells that remain close to the sagittal plane of the telencephalic lobe. Arrows indicate radial migration of differentiated neurons. **C**. Single confocal z-level in the horizontal plane shows ventricular cells photoconverted (arrowhead) in a lateral location of the dorsal rim of the AIS at 2 dpf. **D**. By 4 dpf, a similar horizontal section close to the upper (now ventricular) surface of the telencephalon shows that these cells have generated an anteroposteriorly expanded column of cells. D’. A transverse section through these cells shows that many of these cells are ventricular zone cells contacting the upper ventricular surface of the telencephalic lobe (arrowhead) and projecting radially (arrows) to the lateral pial surface. Schematics show levels of confocal optical sectioning. Scale bars 50 μm. Di: diencephalon; dTel: upper surface of telencephalon; Pal: pallium; Sp: subpallium; Tel: telencephalon.

Our results demonstrate the origin of the OB close to the dorsal rim of the AIS, followed by expansion in telencephalic domains caudal to the OB from 2 dpf to 5 dpf. This expansion is accompanied by the appearance of ventricular zone (VZ) cells and their covering of tela choroidea over the upper surface of the telencephalon. Our analyses of neuropil, OB position, and pallial expansion suggest that the major morphogenetic force driving these changes is an expansion of dorsal telencephalic tissue along the embryo’s anteroposterior axis coupled to a bulging of the posterior telencephalic wall upwards and backwards to meet the roof of the AIS. This possibility suggests that the initial everted form of the telencephalon could occur without the laterally directed outfolding that has been proposed to drive the eversion process. To test this possibility directly and discover whether any outward bending of the lateral telencephalic walls occurs over this period of development, we analyzed VZ morphogenesis by fate mapping using the technique of Kaede photoconversion. We first examined the fate of VZ cells that lie medially in the dorsal rim of the AIS at 2 dpf (Figure 6A). In all cases (*n* = 11) we found that the progeny of these cells are mostly VZ cells that remain close to the median plane at 4 dpf or 5 dpf (Figure 6B-B’). In addition, we observe some neurons that have migrated away from the ventricular zone (Figure 6B-B’). When viewed from the upper surface of the telencephalon, the labelled cells are seen to have expanded into a longitudinal medial column (Figure 6B). We next determined the fate of VZ cells that lie in more lateral positions in the dorsal rim of the AIS (Figure 6C). Their descendants maintain their more lateral location through to 4 dpf and 5 dpf (*n* = 12, Figure 6D-D’). Again, these VZ cells have also expanded to generate a longitudinal column when viewed from the upper telencephalic surface (Figure 6D). These more lateral photoconversions appear to give rise to more migrated neurons. The longitudinal nature of the labelled cell columns at 4 and 5 days demonstrates that the major axis of VZ growth is parallel to the embryo’s AP axis. Our results detect no major mediolateral rearrangements of the VZ cells during this period, although cells leaving the VZ migrate out along radial paths towards the pial surface of the telencephalon (Figure 6B’-D’).

## Discussion

**Figure 7:**
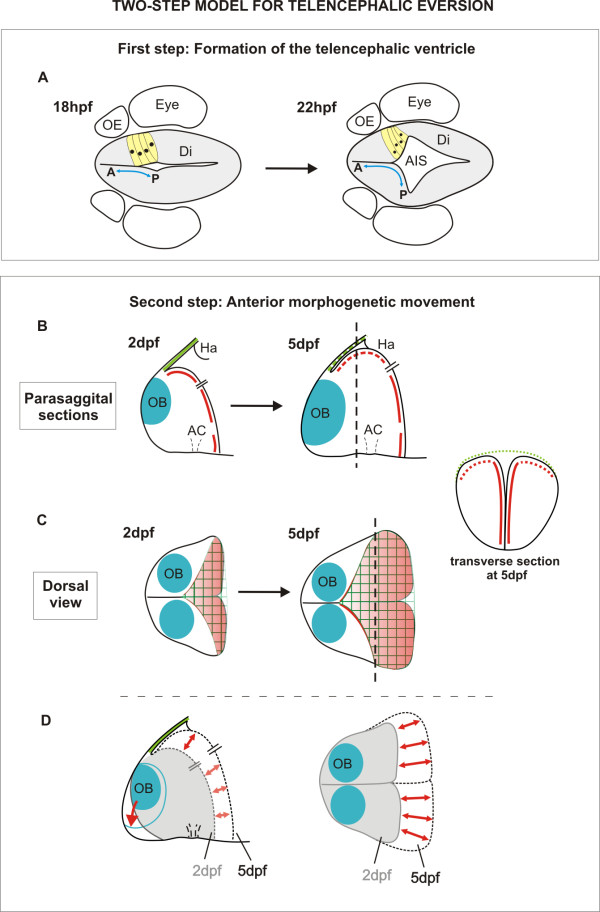
**Two steps in telencephalic morphogenesis.** Summary diagrams illustrate the major morphogenetic movements leading to the everted telencephalon in the zebrafish brain. **A**. First, at around 18 to 22 hpf, an out-pocketing of the ventricular surface forms the anterior intraencephalic sulcus (AIS) with its diamond-shaped roof. This fold forms the posterior wall of the telencephalic lobes and relocates the most posterior telencephalic territory to a more lateral position. **B** and **C**. Next, between 2 dpf and 5 dpf, the pallial domain expands along the AP axis, and the posterior wall of the telencephalon bulges into the ventricular space of the AIS. During this phase, the roof of the AIS (green) also expands along the AP axis to form the tela choroidea. The ventricular surface (red) of the dorsal AIS also bulges into the ventricular space and expands forwards over the upper surface of the telencephalon in close apposition to the tela. These rearrangements of the OB, tela choroidea, and posterior wall of the telencephalon between 2 dpf and 5 dpf are illustrated in diagrammatic parasagittal sections in B and dorsal view in C. The transverse section shows telencephalon has acquired its typical everted morphology at 5dpf. **D**. Parasagittal and dorsal representations to illustrate overall telencephalic growth between 2 and 5 dpf. A; anterior; AC: anterior commissure; AIS: anterior intraencephalic sulcus; Di: diencephalon; Ha: habenula; OB: olfactory bulb; OE: olfactory epithelium; P: posterior.

There is considerable diversity in brain morphology amongst the vertebrates. Much of this diversity probably arises from differential growth of particular CNS regions in the different animal groups but in some cases the morphological differences are more extreme and must be driven by fundamental differences in morphogenetic programmes. One of the most extreme examples of this is the everted morphology of the telencephalon in ray-finned fish. Here we study the morphogenesis of the zebrafish telencephalon and our results highlight two key events in telencephalon morphogenesis that we believe have significant bearing on the development of the everted phenotype (Figure 7). First, the formation of a deep ventricular recess between the telencephalon and the diencephalon, the AIS*,* effectively creates a posterior ventricular wall to the dorsal (pallial) domains of the telencephalic lobes. This process displaces the most posterior territory of the dorsal telencephalon laterally (Figure 7A). Second, from 2 dpf to 5 dpf, expansion of telencephalic territory caudal to the olfactory bulbs along the embryo’s AP axis coincides with the appearance of the pallial ventricular zone and its associated tela choroidea covering on the upper surface of the telencephalon (Figure 7B-D).

### Contributions of the AIS*,* the posterior telencephalic wall, and pallial expansion to the everted phenotype

The AIS is a prominent fold in the early forebrain ventricular surface that lies at the interface of the telencephalic and diencephalic territories, and was described at least 100 years ago [26]. Despite this early documentation, its relevance to the distinctive everted morphology of the telencephalon of ray-finned fish has been largely ignored [9,11,13,20,22]. Our work confirms the generation of the AIS as a primary feature of telencephalic development in the zebrafish, and our data suggest that it may have significant implications in understanding subsequent organization of regional identity within the telencephalon. The potential implications for regional organization stem from our demonstration that AIS formation folds the most posterior territory of the dorsal telencephalic neuroepithelium to a more lateral location (Figure 7A). This folding process generates a posterior ventricular wall to the telencephalon, resulting in anteroposterior positional values becoming realigned along the mediolateral axis (Figure 7A). If specification of pallial regional identity is established before the AIS is initiated, then the neuroepithelium that is located at the most posterior region of the dorsal telencephalic anlage will become placed first more laterally, and this is supported by our analysis of OB and pallial markers.

Following formation of the AIS and posterior telencephalic wall, the next significant morphogenetic process is the expansion of the pallium domains between days 2 and 5 (Figure 7B-D). During this phase, fate-mapping data and analysis of tela choroidea expansion show that the major axis of neuroepithelial growth is aligned along the embryo’s AP axis. Our data are reminiscent of those of Droogleever Fortuyn [31], who suggested that eversion of the stickleback dorsal telencephalon includes a phase of forward-directed growth (see his Figures 10 and 11). We believe that our data are best interpreted as the pallial expansion being accompanied by a bulging convexity of the posterior telencephalic wall pushing the dorsal aspects of the posterior telencephalic wall up against the roof of the AIS. We believe that this is the key aspect of telencephalic morphogenesis that initiates the everted phenotype by pushing the ventricular surface of the posterior telencephalic wall up towards the dorsal surface. This generates the narrow ventricular space and tela choroidea that begin to enclose the upper surface of the telencephalon. Our fate mapping suggests that there is very little mediolateral reorganization of the ventricular surface during this period. Our data, therefore, do not support the widely held model that the everted phenotype results from an outward bending of the lateral walls of the telencephalon.

#### Comparisons with previous models of eversion

Although the characteristics of an everted telencephalon are clear [11,12] (see Figure 1), the process that generates such morphology (eversion) has been difficult to define. Based mainly on the morphology of the adult telencephalon, eversion has been thought to consist of a lateral outfolding of the dorsal region of the neural tube, such that the dorsal telencephalon (pallium) folds laterally over the ventral telencephalon and the roof plate expands mediolaterally [11,15,17-23]. Implicit in this model is the assumption that the anterior to posterior regional identity of the ventricular tissue undergoing eversion should be unaffected. However, there have been no previous experimental studies that directly address tissue and cell morphogenesis in living embryos. Based on our current work, we can now ask how the zebrafish data compares with the conventional view.

Our data show that early telencephalic morphogenesis does include a lateral outfolding of the neuroepithelium but that this folding occurs at the telencephalic-diencephalic interface to generate the AIS and folds the posterior telencephalon to a more lateral position (Figure 7A). Unlike the folding event in the conventional model of eversion, this folding process does not bring the medial ventricular surface of the telencephalon to the upper surface but instead generates a posterior ventricular wall to the telencephalon. Our data suggest that from approximately 48 hpf the posterior ventricular wall bulges up and into the AIS. This brings the posterior ventricular wall of the telencephalon into close proximity with the roof of the AIS, and this is the first step that causes a small area of ventricle to lie over the upper surface of the telencephalon. The dorsal telencephalon then grows along the AP axis, increasing the amount of ventricle on the upper surface of the telencephalon and stretching the roof of the AIS forward to become the tela choroidea (Figure 7B-D). These data between days 1 and 5 are not consistent with the conventional view that eversion is driven by an outward bending of the lateral walls of the telencephalon. However, after 5 dpf, the everted morphology could well be refined to acquire the final morphology observed in the adult by a variety of processes that could include differential growth, further folding of the neuroepithelium, and cell migration.

Our data are not the first to suggest that the commonly held view of eversion needs modification. Previous studies of the organization of telencephalic areas and their connections in adult goldfish found that this did not fit the predictions of the classic model for telencephalic eversion [9,10]. Yamamoto and colleagues [10] proposed a new eversion model based only on their regional analyses in the adult. A major component of their hypothesis is a ‘caudolateral’ folding of the embryonic pallial neuroepithelium that could be consistent with the movements we describe generating the AIS. Our analysis demonstrates that this outfolding precedes the phase of eversion that brings the ventricular cells and their covering of tela choroidea onto the superficial surface of the telencephalon, whereas their hypothesis implies that it follows this event (see their Figure 4A). Interestingly, their study suggests, and our data demonstrate, that the mediolateral outfolding is more complex at the posterior pole of the telencephalon than the anterior pole. In contrast to the rearrangements at the posterior pole of the telencephalon, our data do not support the Yamamoto *et al.* view that a relatively simple lateral outfolding of the anterior pallium occurs. However, it is, of course, possible that such folding could occur beyond day 5 of development.

Our results also show that there is more than one way to obtain the characteristic organization of radial glia often used as one criterion in support of the conventional model of eversion (e.g. [11]). Coronal sections of adult teleost telencephalon show radial glia with cell bodies adjacent to the upper everted surface and their radial processes curving laterally to the pial surface. Our analysis shows that a similar organization of cell shapes is generated in the neuroepithelium during formation of the AIS, but at this time the fold in the neuroepithelium is in the horizontal plane, not the coronal plane. However, the upwards and backwards bulging expansion of the posterior wall of the telencephalon towards the tela choroidea of the AIS between 2 and 5 days would reorient this cellular arrangement from the horizontal plane to the coronal plane.

#### Mechanisms to drive two-step morphogenesis

The generation of the AIS must be driven by local regulation of neuroepithelial cytoskeleton to control cell shape changes required to fold the epithelium. Cells destined to lie at the lateral extremes of the AIS must shorten and constrict their apical profile. The second step of our model involves the expansion of pallial territory along the AP axis to increase the distance between the OB primordia and the dorsal diencephalon. This appears to be coupled to an increase in the convexity of the dorsal aspects of the posterior telencephalic wall. The posterior wall appears to bulge back and up towards the roof of the AIS. The AP vector of ventricular expansion could either be driven by oriented divisions along the AP axis or contain an element of cell intercalation that expands clones along a particular orientation.

## Conclusion

Our data describing telencephalic morphogenesis challenge the conventional model that eversion is simply caused by a laterally directed outfolding of the telencephalic neuroepithelium. This result may have significant bearing on understanding eventual organization of the adult fish telencephalon.

## Methods

### Fish stocks and maintenance

Zebrafish strains (*Danio rerio*, Cyprinidae, ray-finned fish) were maintained and bred following standard procedures. AB wild type, Tg(elavl3:eGFP)^zf8^[32], Et(gata2:GFP)^bi105^, Tg(1.4dlx5a-dlx6a:GFP)^ot1^[33], Tg(−10lhx2a:EGFP)^zf176^[34], Et(CLG-YFP)^smb8^[35], Tg(β-actin:HRAS-EGFP)^vu119^[25], and Tg(−8.0cldnb:lynEGFP)^zf106^ were used. Embryos were raised at 28.5°C and staged according to standard methods [36]. To prevent pigment formation, 0.003% w/v phenylthiocarbamide (PTU, Sigma) was added to the fish water at 24 hpf (hours post fertilization).

### Synthesis and injection of mRNA

The cDNAs used in this work for RNA synthesis were cloned previously in pCS2 (*kaede*, [37]; *pard3/asip-egfp*, [25]) and mRNA was generated using a Message Machine RNA synthesis kit (Ambion) according to manufacturer’s instructions. Embryos were injected with 2–3 nl of 0.1 pg/μl mRNA at the one to four cell stage.

### Time-lapse imaging

Confocal time-lapse imaging was performed using Tg(β-actin:HRAS-EGFP)^vu119^ line. Once anaesthetized, embryos were embedded in low melting point agarose and imaged from a dorsal view using water immersion objectives. Embryos were maintained at 28.5°C in an environmental chamber and z-stacks were collected at intervals of 5 to 8 min. Imaging was started at around 14 hpf to 16 hpf and usually continued for 10 to 12 hours.

### BrdU pulses

Embryos were transferred to a small dish containing a solution of bromodeoxyuridine (BrdU, 5 mg/ml) diluted in fish water with 15% dimethyl sulfoxide (DMSO, Sigma). The dish was kept on ice for 20 min and then transferred to 28.5°C (20 min). After recovering from treatment (5 min) in fish water, embryos were fixed in 4% paraformaldehyde and immunostained.

### Antibody labelling and *in situ* hybridization procedures

For immunostaining, embryos were fixed in 4% paraformaldehyde and stained as whole mounts, following standard procedures [38]. Antibodies and dilutions used were mouse anti-BrdU (1:200, Sigma), rabbit anti-green fluorescent protein (GFP; 1:1000, Torrey Pines Biolabs), rabbit anti-glial fibrillary acid protein (GFAP; 1:400, Dako), zona occludens 1 (ZO1; 1:300, Invitrogen) mouse anti-acetylated tubulin antibody (1:250, Sigma).

For *in situ* hybridization, antisense RNA probes were generated using digoxigenin RNA labelling kits (Boehringer-Mannheim). Whole-mount *in situ* hybridization was performed as previously described [39].

### Kaede fate mapping

Embryos were anaesthetized and mounted in low melting point agarose. A small number of cells expressing Kaede were photoconverted from green to red [37] using the 405 nm line on a Leica SP5 confocal microscope. Images of the initial photoconverted region were taken. Embryos were then released from their agarose mount and maintained at 28.5°C until two or three days later, when they were reimaged to assess the fate of the photoconverted cells.

## Abbreviations

AC: anterior commissure; AIS: anterior intraencephalic sulcus; Di: diencephalon; dTel: dorsal telencephalon; DVDT: dorsoventral diencephalic tract; E: epithalamus; Ha: habenula; Hy: hypothalamus; Me: mesencephalon; OB: olfactory bulb; OC: optic chiasm; OE: olfactory epithelium; ON: olfactory nerve; P: pineal; Pal: pallium; PC: posterior commissure; POa: preoptic area; POC: postoptic commissure; PTW: posterior telencephalic wall; rp: roof plate; SM: stria medullaris; SOT: supraoptic tract; Sp: subpallium; TAC: tract of the anterior commissure; tc: tela choroidea; Tel: telencephalon; TPC: tract of the posterior commissure; TPOC: tract of the postoptic commissure; V: ventricle; VZ: ventricular zone.

## Competing interests

The authors declare that they have no competing interests.

## Authors’ contributions

MF and PB carried out the *in situ* hybridization and antibody analyses of telencephalic organization and both participated in the Kaede fate-mapping experiments and drafting of manuscripts. The contributions of MF and BP should be considered equal. MF made the time-lapse analyses of AIS formation. PN and TS generated the Et(CLG-YFP)^smb8^ and Et(gata2:GFP)^bi105^ lines. SW and JC oversaw the whole project and contributed to manuscript writing. All authors read and approved the final manuscript.

## Authors’ information

MF has a joint appointment with the Department of Cell and Molecular Biology, University of A Coruña, 15008 A Coruña, Spain.

## Supplementary Material

Additional file 1 **Movie 1.** Confocal time-lapse showing forebrain AIS morphogenesis and formation of the posterior wall of the telencephalic lobes in a Tg(β-actin:HRAS-EGFP)^vu119^ embryo. Five z-levels seen from the dorsal view are shown (most superficial to the left) to illustrate that AIS opens first ventrally. Anterior is up. Click here for file

Additional file 2 **Figure S1.** Olfactory bulb markers and fate mapping suggest that origin of OB is close to the dorsal rim of the AIS. A-D. Whole-mount *in situ* hybridizations for *lhx1a* at different stages from 1 dpf to 5 dpf, showing that the distance between the olfactory bulb domain (arrowhead) and the AIS (dotted line) increases between days 1 and 5. All lateral views with anterior to the left. E and F. YFP expression in the olfactory bulb of the transgenic Et(CLG-YFP)^smb8^ embryos also becomes increasingly distant from the AIS (white dotted line) between 2 and 5 dpf. Neuropil is counterstained with an antibody recognizing acetylated tubulin (red). All images are lateral views, anterior to the left. G to J. Photoconversion of Kaede-expressing cells close to the dorsal rim of the AIS at 1 dpf reveals that descendants from these cells populate the olfactory bulb at 5 dpf. G. Drawing illustrating the three regions of the telencephalon targeted by Kaede photoconversion for fate mapping at 1 dpf (T1-3, lateral view). H. Summary distribution in lateral views at 5 dpf of cells photoconverted in the regions illustrated in G. All data derived from confocal z-stacks of the whole telencephalon. I-J. Example of photoconversion and fate from T1. I is a lateral non-confocal view of the photoconverted cells (blue arrow) at 1 dpf in region T1. J is a single confocal section taken at 5 dpf at the level illustrated by the red line in H. Photoconverted cells are red and non-converted cells remain green. Scale bars 50 μm. AC: anterior commissure; E: epithalamus; Ha: habenula; Hy: hypothalamus; OB: olfactory bulb; SM: stria medullaris; SOT: supraoptic tract; Tel: telencephalon; V: ventricle. Click here for file

Additional file 3 **Movie 2.** Three-dimensional reconstruction from confocal z-stack of the telencephalon of a 2 dpf embryo stained for ZO-1 (red) to reveal ventricular surface and GFAP (green) to show depth of neuroepithelium. As it rotates, note that the ventricular surface is only present on the medial and posterior surfaces of the telencephalic lobes, but not on the upper telencephalic surface at this time. Anterior is to the left, initial view is from dorsal aspect and this rotates to give first a posterior and then lateral view. Roof of the AIS has been dissected away to allow better resolution of the ventricular surface of the telencephalon. Click here for file

Additional file 4 **Figure S2.** Attachment of the tela choroidea in relation to the olfactory bulb in other fishes. Parasagittal section from *Amia calva* (top) and *Ameiurus nebulosus* (bottom) showing the attachment of the tela choroidea (t.c.) to a point just caudal to the olfactory bulb (b.o.). Although the tela attachments are very close to the olfactory bulb, a small pallial region could be interposed between the two. The organization shown in these adult fish is consistent with our data that the origin of the olfactory bulb is very close to the origin of the tela in the roof of the AIS. Illustration modified from [12]. Click here for file
