# Supplementary figures and images for: Morphogenesis underlying the development of the everted teleost telencephalon
Source: Neural Dev. 2012 Sep 18;7:32. doi: 10.1186/1749-8104-7-32 (PMC3520737; doi:10.1186/1749-8104-7-32)

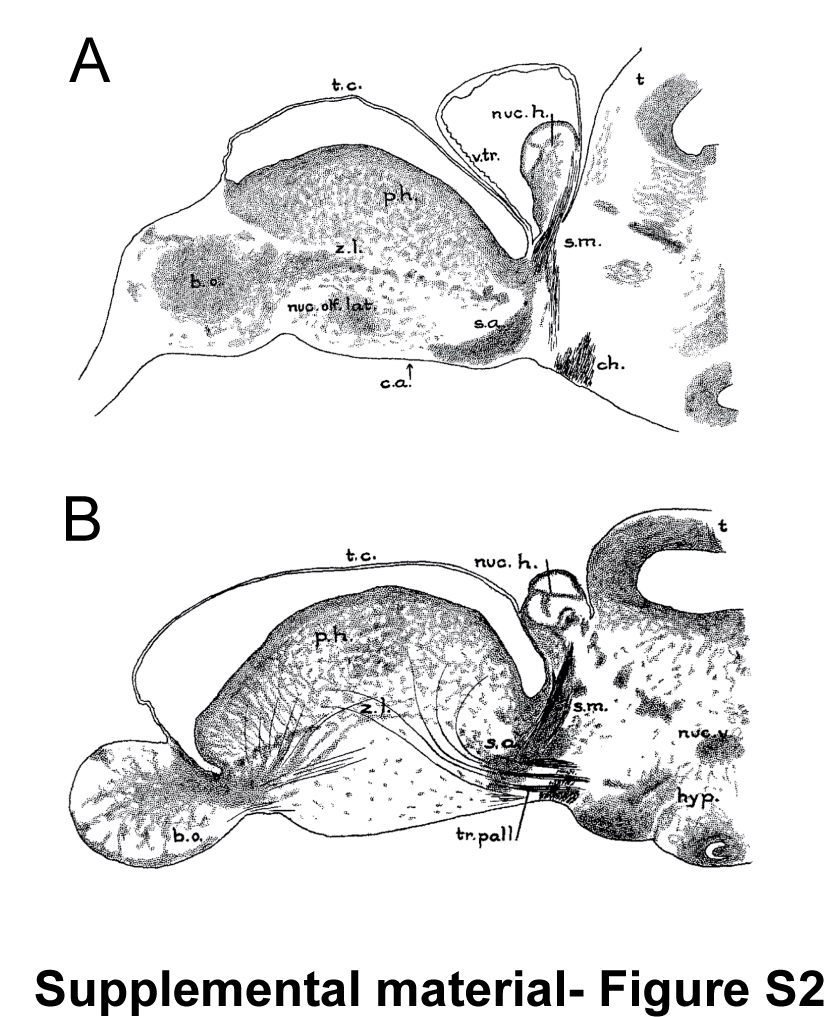

Supplement: Additional file 4 — Figure S2. Attachment of the tela choroidea in relation to the olfactory bulb in other fishes. Parasagittal section from Amia calva (top) and Ameiurus nebulosus (bottom) showing the attachment of the tela choroidea (t.c.) to a point just caudal to the olfactory bulb (b.o.). Although the tela attachments are very close to the olfactory bulb, a small pallial region could be interposed between the two. The organization shown in these adult fish is consistent with our data that the origin of the olfactory bulb is very close to the origin of the tela in the roof of the AIS. Illustration modified from [12]. [file 1749-8104-7-32-S4.jpeg]
