# Supplementary material for: Erratum to: Morphogenesis underlying the development of the everted teleost telencephalon
Source: Neural Dev. 2015 Oct 2;10:22. doi: 10.1186/s13064-015-0048-4 (PMC4605123; doi:10.1186/s13064-015-0048-4)
Supplement: Additional file 2: Figure S1. — Olfactory bulb markers and fate mapping suggest that origin of OB is close to the dorsal rim of the AIS. A-D. Whole-mount in situ hybridizations for lhx1a at different stages from 1 dpf to 5 dpf, showing that the distance between the olfactory bulb domain (arrowhead) and the AIS (dotted line) increases between days 1 and 5. All lateral views with anterior to the left. E and F. YFP expression in the olfactory bulb of the transgenic Et(CLG-YFP)smb8 embryos also becomes increasingly distant from the AIS (white dotted line) between 2 and 5 dpf. Neuropil is counterstained with an antibody recognizing acetylated tubulin (red). All images are lateral views, anterior to the left. G to J. Photoconversion of Kaede-expressing cells close to the dorsal rim of the AIS at 1 dpf reveals that descendants from these cells populate the olfactory bulb at 5 dpf. G. Drawing illustrating the three regions of the telencephalon targeted by Kaede photoconversion for fate mapping at 1 dpf (T1-3, lateral view). H. Summary distribution in lateral views at 5 dpf of cells photoconverted in the regions illustrated in G. All data derived from confocal z-stacks of the whole telencephalon. I-J. Example of photoconversion and fate from T1. I is a lateral non-confocal view of the photoconverted cells (blue arrow) at 1 dpf in region T1. J is a single confocal section taken at 5 dpf at the level illustrated by the red line in H. Photoconverted cells are red and non-converted cells remain green. Scale bars 50 μm. AC: anterior commissure; E: epithalamus; Ha: habenula; Hy: hypothalamus; OB: olfactory bulb; SM: stria medullaris; SOT: supraoptic tract; Tel: telencephalon; V: ventricle. (DOC 1779 kb) [file 13064_2015_48_MOESM2_ESM.doc]

**
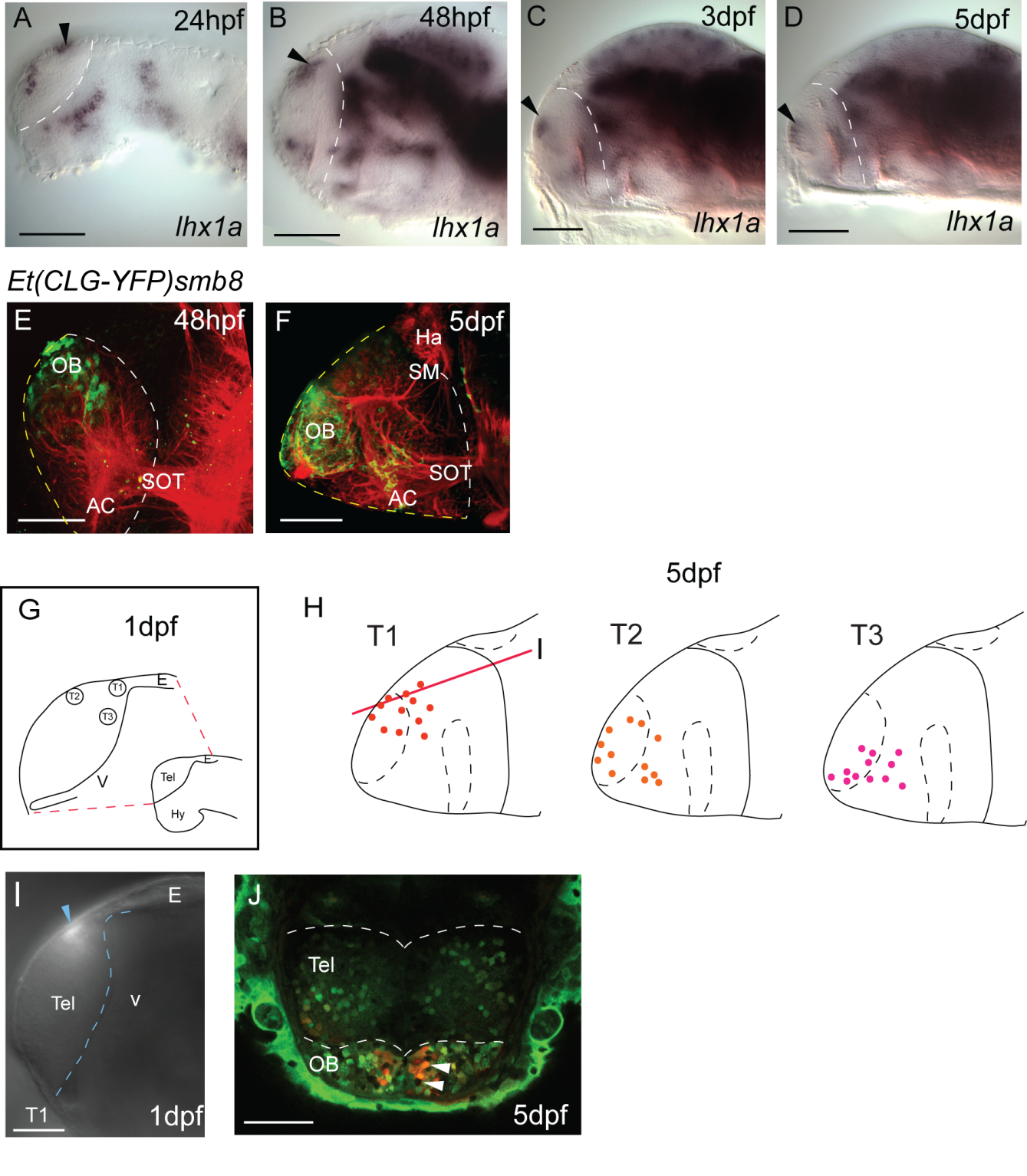
**

**Additional file 2. Figure S1.** Olfactory bulb markers and fate mapping suggest that origin of OB is close to the dorsal rim of the AIS. A-D. Whole-mount in situ hybridizations for lhx1a at different stages from 1 dpf to 5 dpf, showing that the distance between the olfactory bulb domain (arrowhead) and the AIS (dotted line) increases between days 1 and 5. All lateral views with anterior to the left. E and F. YFP expression in the olfactory bulb of the transgenic Et(CLG-YFP)smb8 embryos also becomes increasingly distant from the AIS (white dotted line) between 2 and 5 dpf. Neuropil is counterstained with an antibody recognizing acetylated tubulin (red). All images are lateral views, anterior to the left. G to J. Photoconversion of Kaede-expressing cells close to the dorsal rim of the AIS at 1 dpf reveals that descendants from these cells populate the olfactory bulb at 5 dpf. G. Drawing illustrating the three regions of the telencephalon targeted by Kaede photoconversion for fate mapping at 1 dpf (T1-3, lateral view). H. Summary distribution in lateral views at 5 dpf of cells photoconverted in the regions illustrated in G. All data derived from confocal z-stacks of the whole telencephalon. I-J. Example of photoconversion and fate from T1. I is a lateral non-confocal view of the photoconverted cells (blue arrow) at 1 dpf in region T1. J is a single confocal section taken at 5 dpf at the level illustrated by the red line in H. Photoconverted cells are red and non-converted cells remain green. Scale bars 50 μm. AC: anterior commissure; E: epithalamus; Ha: habenula; Hy: hypothalamus; OB: olfactory bulb; SM: stria medullaris; SOT: supraoptic tract; Tel: telencephalon; V: ventricle.
